# Supplementary material for: Modeling glioblastoma heterogeneity as a dynamic network of cell states
Source: Mol Syst Biol. 2021 Sep 16;17(9):e10105. doi: 10.15252/msb.202010105 (PMC8444284; doi:10.15252/msb.202010105)
Supplement: Supplementary file 6 — Source Data for Figure 5 [file MSB-17-e10105-s004.zip › Figure5A_sourcedata/GSEA_3071/nosigdb_stateV.GseaPreranked.1622795159954/NB_KI_CLUSTER5.html]

Details for gene set NB\_KI\_CLUSTER5[GSEA]

|  || Dataset | state43071 |
| Phenotype | NoPhenotypeAvailable |
| Upregulated in class | na\_pos |
| GeneSet | NB\_KI\_CLUSTER5 |
| Enrichment Score (ES) | 0.77492136 |
| Normalized Enrichment Score (NES) | 4.036318 |
| Nominal p-value | 0.0 |
| FDR q-value | 0.0 |
| FWER p-Value | 0.0 |
Table: GSEA Results Summary

  

Fig 1: Enrichment plot: NB\_KI\_CLUSTER5      
 Profile of the Running ES Score & Positions of GeneSet Members on the Rank Ordered List

  

| PROBE | GENE SYMBOL | GENE\_TITLE | RANK IN GENE LIST | RANK METRIC SCORE | RUNNING ES | CORE ENRICHMENT || 1 | HIST1H4C |  |  | 1 | 2.442 | 0.0259 | Yes |
| 2 | ATAD2 |  |  | 2 | 2.214 | 0.0496 | Yes |
| 3 | HMGB2 |  |  | 5 | 2.011 | 0.0707 | Yes |
| 4 | HIST1H1C |  |  | 7 | 1.976 | 0.0916 | Yes |
| 5 | PCNA |  |  | 10 | 1.908 | 0.1117 | Yes |
| 6 | HIST1H1E |  |  | 15 | 1.830 | 0.1304 | Yes |
| 7 | DHFR |  |  | 18 | 1.795 | 0.1493 | Yes |
| 8 | PCLAF |  |  | 27 | 1.720 | 0.1661 | Yes |
| 9 | HIST2H2AC |  |  | 31 | 1.681 | 0.1835 | Yes |
| 10 | BRCA1 |  |  | 46 | 1.592 | 0.1978 | Yes |
| 11 | HELLS |  |  | 48 | 1.588 | 0.2146 | Yes |
| 12 | MCM3 |  |  | 49 | 1.586 | 0.2316 | Yes |
| 13 | EZH2 |  |  | 55 | 1.534 | 0.2470 | Yes |
| 14 | GINS2 |  |  | 57 | 1.528 | 0.2631 | Yes |
| 15 | MXD3 |  |  | 62 | 1.500 | 0.2784 | Yes |
| 16 | CDK1 |  |  | 69 | 1.488 | 0.2932 | Yes |
| 17 | SMC2 |  |  | 73 | 1.466 | 0.3082 | Yes |
| 18 | CHAF1A |  |  | 77 | 1.441 | 0.3231 | Yes |
| 19 | UBE2T |  |  | 79 | 1.432 | 0.3382 | Yes |
| 20 | FBXO5 |  |  | 82 | 1.422 | 0.3530 | Yes |
| 21 | CLSPN |  |  | 84 | 1.417 | 0.3680 | Yes |
| 22 | CIP2A |  |  | 86 | 1.410 | 0.3829 | Yes |
| 23 | MCM7 |  |  | 88 | 1.400 | 0.3976 | Yes |
| 24 | DEK |  |  | 94 | 1.384 | 0.4115 | Yes |
| 25 | CENPM |  |  | 114 | 1.337 | 0.4221 | Yes |
| 26 | SMC4 |  |  | 127 | 1.320 | 0.4339 | Yes |
| 27 | LIG1 |  |  | 145 | 1.284 | 0.4444 | Yes |
| 28 | GMNN |  |  | 146 | 1.280 | 0.4580 | Yes |
| 29 | NCAPD2 |  |  | 157 | 1.252 | 0.4695 | Yes |
| 30 | MIS18BP1 |  |  | 159 | 1.247 | 0.4826 | Yes |
| 31 | CENPK |  |  | 162 | 1.241 | 0.4955 | Yes |
| 32 | DNMT1 |  |  | 166 | 1.237 | 0.5082 | Yes |
| 33 | NASP |  |  | 180 | 1.212 | 0.5186 | Yes |
| 34 | GGH |  |  | 182 | 1.207 | 0.5313 | Yes |
| 35 | DBF4 |  |  | 189 | 1.197 | 0.5430 | Yes |
| 36 | CDKN2D |  |  | 190 | 1.196 | 0.5558 | Yes |
| 37 | CDT1 |  |  | 194 | 1.185 | 0.5679 | Yes |
| 38 | FANCA |  |  | 199 | 1.173 | 0.5796 | Yes |
| 39 | ZWINT |  |  | 204 | 1.166 | 0.5913 | Yes |
| 40 | PRC1 |  |  | 211 | 1.161 | 0.6026 | Yes |
| 41 | ARL6IP1 |  |  | 212 | 1.158 | 0.6150 | Yes |
| 42 | MCM5 |  |  | 243 | 1.127 | 0.6213 | Yes |
| 43 | SGO2 |  |  | 244 | 1.126 | 0.6333 | Yes |
| 44 | ECT2 |  |  | 295 | 1.071 | 0.6352 | Yes |
| 45 | CKAP2 |  |  | 312 | 1.053 | 0.6433 | Yes |
| 46 | DUT |  |  | 314 | 1.050 | 0.6544 | Yes |
| 47 | PRR11 |  |  | 317 | 1.048 | 0.6652 | Yes |
| 48 | CDCA7 |  |  | 330 | 1.038 | 0.6740 | Yes |
| 49 | DDX11 |  |  | 331 | 1.038 | 0.6851 | Yes |
| 50 | NUDT1 |  |  | 417 | 0.936 | 0.6788 | Yes |
| 51 | KPNA2 |  |  | 435 | 0.919 | 0.6853 | Yes |
| 52 | LBR |  |  | 438 | 0.916 | 0.6947 | Yes |
| 53 | RACGAP1 |  |  | 443 | 0.912 | 0.7037 | Yes |
| 54 | SHCBP1 |  |  | 465 | 0.900 | 0.7093 | Yes |
| 55 | HMGN2 |  |  | 466 | 0.900 | 0.7189 | Yes |
| 56 | UBE2S |  |  | 514 | 0.854 | 0.7191 | Yes |
| 57 | PLK1 |  |  | 526 | 0.850 | 0.7260 | Yes |
| 58 | BIRC5 |  |  | 541 | 0.842 | 0.7323 | Yes |
| 59 | TCF19 |  |  | 542 | 0.842 | 0.7413 | Yes |
| 60 | CEP152 |  |  | 547 | 0.834 | 0.7495 | Yes |
| 61 | CDC25B |  |  | 566 | 0.820 | 0.7548 | Yes |
| 62 | HMGB1 |  |  | 583 | 0.810 | 0.7604 | Yes |
| 63 | DIAPH3 |  |  | 585 | 0.808 | 0.7688 | Yes |
| 64 | ANP32E |  |  | 722 | 0.739 | 0.7506 | Yes |
| 65 | TEX30 |  |  | 730 | 0.730 | 0.7571 | Yes |
| 66 | CHEK1 |  |  | 779 | 0.709 | 0.7555 | Yes |
| 67 | TRIP13 |  |  | 804 | 0.698 | 0.7583 | Yes |
| 68 | H2AFZ |  |  | 851 | 0.677 | 0.7568 | Yes |
| 69 | MZT1 |  |  | 917 | 0.652 | 0.7513 | Yes |
| 70 | H2AFV |  |  | 933 | 0.647 | 0.7553 | Yes |
| 71 | KIF20B |  |  | 934 | 0.647 | 0.7622 | Yes |
| 72 | PTTG1 |  |  | 936 | 0.647 | 0.7689 | Yes |
| 73 | CKS2 |  |  | 966 | 0.634 | 0.7701 | Yes |
| 74 | RANBP1 |  |  | 999 | 0.620 | 0.7706 | Yes |
| 75 | TUBA1B |  |  | 1012 | 0.617 | 0.7749 | Yes |
| 76 | ZWILCH |  |  | 1048 | 0.609 | 0.7747 | No |
| 77 | NUCKS1 |  |  | 1178 | 0.570 | 0.7561 | No |
| 78 | ANP32B |  |  | 1187 | 0.566 | 0.7606 | No |
| 79 | SPDL1 |  |  | 1397 | 0.507 | 0.7259 | No |
| 80 | CKS1B |  |  | 1502 | 0.479 | 0.7111 | No |
| 81 | TUBB4B |  |  | 1659 | 0.444 | 0.6859 | No |
| 82 | CENPW |  |  | 1959 | 0.395 | 0.6327 | No |
| 83 | RHEB |  |  | 2192 | 0.363 | 0.5921 | No |
| 84 | BUB3 |  |  | 2806 | 0.288 | 0.4776 | No |
Table: GSEA details [plain text format]

  

Fig 2: NB\_KI\_CLUSTER5: Random ES distribution      
 Gene set null distribution of ES for **NB\_KI\_CLUSTER5**
